# Prostatic Abscess Secondary to Staphylococcus haemolyticus and Escherichia coli: A Case Report

**DOI:** 10.7759/cureus.40406

**Published:** 2023-06-14

**Authors:** Marco Bermudez, Steven B Epstein, Nehemias Guevara, Laura Pedraza, Michelle Dahdouh, Ihab Awad

**Affiliations:** 1 Internal Medicine, SBH Health System, New York, USA; 2 Interventional Radiology, SBH Health System, New York, USA; 3 Infectious Disease, SBH Health System, New York, USA; 4 Urology, SBH Health System, New York, USA

**Keywords:** ultrasound-guided drainage, s: sepsis, staphylococcus haemolyticus, uti: urinary tract infection, prostate abscess

## Abstract

A prostate abscess is a rare clinical entity with an incidence of 0.2%-0.5% in males. No case reports exist of *Staphylococcus haemolyticus *as an etiologic bacterial agent. We report a 59-year-old man with a past medical history of poorly controlled diabetes mellitus and benign prostatic hyperplasia who was hospitalized due to urosepsis and obstruction. A prostatic abscess was discovered and initially treated with intravenous vancomycin and ertapenem. Clinical improvement was apparent within two days following transrectal prostatic abscess drainage. Four weeks of intravenous antibiotics followed. Prostatic abscess cultures grew* Staphylococcus haemolyticus* and *Escherichia coli* following tube deployment. To the best of our knowledge, this is the first case reporting *Staphylococcus haemolyticus* as an organism in a prostate abscess. We regard this as another example of the rising incidence of gram-positive organisms in prostatic abscesses in the post-antibiotic era.

## Introduction

A prostatic abscess (PA) is mainly caused by gram-negative organisms such as *Escherichia coli*, *Klebsiella pneumoniae*, *Enterococcus* species, *Pseudomonas aeruginosa*, and *Burkholderia pseudomallei*. The most common is *Escherichia coli* [1]. Acute and chronic bacterial prostatitis are knowingly colonized by *Escherichia coli* and *Staphylococcus haemolyticus* [2,3]. However, the latter has not been implicated as an etiological agent of prostate abscess in the medical literature. This case emphasizes the need to consider gram-positive organisms such as *Staphylococcus haemolyticus* as causative organisms for prostate abscess formation, as the increase in* Staphylococcus* species infections causing prostate abscesses in the last decade should add consideration for initial empiric broad-spectrum gram-positive and gram-negative antibiotic coverage, especially in immunocompromised patients. Clinicians should be aware of the increase in *Staphylococcus* species causing PAs. Broad-spectrum empiric antibiotic coverage for PA should include gram-positive and gram-negative broad-spectrum coverage before tailoring antibiotics accordingly with procedural drainage cultures. Management of PA is not standardized, as few cases are reported [4]. This case report was prepared following the CARE (CAse REport) guidelines [5].

This article was previously presented as a poster abstract at the 2022 NYACP Annual Scientific Meeting on November 5, 2022, and at the 2023 ACP Annual Internal Medicine Meeting National Poster Competition on April 28, 2023.

## Case presentation

A 59-year-old man with a medical history of hypertension, poorly controlled diabetes mellitus Type 2 (DM2), and benign prostatic hyperplasia (BPH) who had required intermittent catheterizations for repeated bouts of urinary retention. The patient's home medications included amlodipine 5 mg daily, tamsulosin 0.4 mg daily, and metformin 500 mg BID to control his DM2. The patient did not follow up regularly with his primary care physician and was not adherent to his medications, diet, and exercise regimen. The patient was initially admitted due to acute urinary retention (AUR). 

He also reported malaise and vague lower abdominal discomfort. His temperature was 36.6 degrees Celsius (97.8 degrees Fahrenheit), blood pressure 132/72 mmHg, heart rate 105 beats per minute, and oxygen saturation 96% on room air. He was alert, awake, and oriented. A physical exam revealed suprapubic tenderness and a palpable, distended bladder. Initial laboratories (Table 1) showed glycosylated hemoglobin (HBA1C) of 14%, with leukocytosis of 22,700/ul and neutrophilia of 86.6%; furthermore, normocytic normochromic anemia with a hemoglobin of 11.3g/dL, hyperglycemia of 456 mg/dl with negative serum ketones, a urinalysis with positive leukocyte esterase, white blood cell count (WBC) of >182/high-power field, many bacterias, and many WBC clumps. The remaining initial lab results were unremarkable.

**Table 1:** Initial diagnostic workup of the patient and its trend during hospitalization HbA1C: hemoglobin A1C; WBC: white blood cells; ALT: alanine transaminase; AST: aspartate transaminase

	Day one	Day two	Day three	Day four	Day five	Day seven	Day nine	Day ten	Day twenty
Sodium (mEq/L)	137				136				136
Potassium (mEq/L)	3.8				3.7				4.1
Creatinine (mg/dL)	0.9				0.7				0.5
Glucose (mg/dL)	456				201				214
HbA1C (%)							11.7		
Albumin (gm/dL)	2.6				1.9				
ALT (IU/L)	36				48				
AST (IU/L)	21				37				
Alkaline phosphatase (IU/L)	234				203				
Hemoglobin (gm/dl)	11.3				10.2				10.2
WBC	23.0	29.2	29.8	25.9	20.1	22.8	19.3	20.6	9.1
Neutrophils (%)	86	82.1	82.9	80.9	87.0	77.5	72.5	71.3	52.6

Intravenous (IV) ceftriaxone was administered at 1 gram daily to manage a complicated urinary tract infection (UTI). Hyperglycemia was managed with a sliding insulin scale. Balloon-expanded transurethral (Foley) catheter was deployed. Ultrasound examination demonstrated no hydronephrosis or nephrolithiasis. 

**Figure 1:**
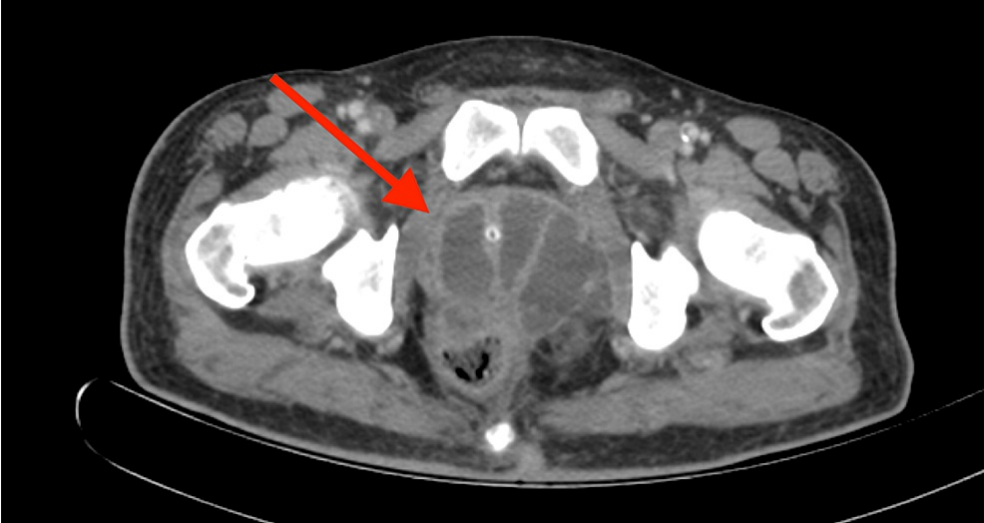
Computed tomography demonstrates multiloculated prostate abscess and transurethral segment of bladder catheter

On the second day of hospitalization, blood cultures were negative, while urine cultures grew *Klebsiella pneumoniae* with positive extended-spectrum beta-lactamase (ESBL) susceptible to ertapenem. Therefore IV ceftriaxone was changed to IV ertapenem 1 gram per day. Fever and leukocytosis persisted despite four days of tailored IV antibiotics, according to the final results of the urine culture. On day seven of hospitalization, multiphase computed tomography (CT) demonstrated multiloculated, septated PA, which measured 6.2 x 6.8 cm (Figure 1). Digital examination was positive for a fluctuating and painful prostate.

Interventional radiology (IR) was consulted. Medicine, urology, infectious disease, and IR concurred that abscess drainage should be undertaken. The patient received several tap water enemas. 

**Figure 2:**
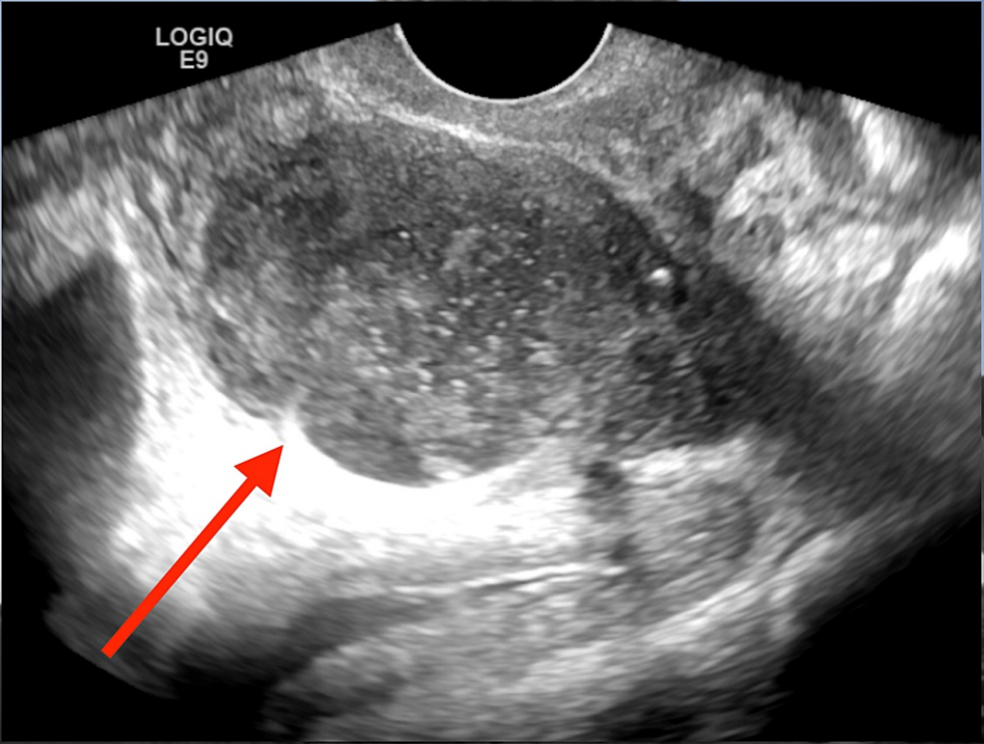
Transrectal ultrasound; probe gently angled and rotated prior to entry with an 18-gauge trocar needle

Transrectal ultrasound (TRUS) guided needle aspiration and drainage was performed a day later. The patient was on a stretcher in the IR suite, turned left lateral decubitus with knees bent. Procedure was performed using transrectal ultrasound only. Fluoroscopy and contrast were not deemed necessary. A guiding clip was attached to a transrectal probe. An 18-gauge trocar needle was advanced into an interseptal, compartmented abscess, then retracted, then into another compartment, and then into another. Septa were also purposefully penetrated with this needle. Sharp inner stylet was removed, and a stiff (Amplatz) wire was repeatedly advanced and pulled back while the ultrasound probe was slightly rotated. These maneuvers effected free communication of all abscess compartments. Eighty milliliters of frankly purulent fluid were initially encountered (Figure 2). A 10 French tube exited through his anorectal canal. Pigtail loop was locked within the abscess and not sewed in place. 

 

Prompt and dramatic clinical improvement followed from day 8 to day 13 of hospitalization. WBCs eventually normalized, perineal pain disappeared, and sepsis resolved. Additional urologic surgery was not necessary.

On day 13 of hospitalization, cultures from tube samplings yielded *Escherichia coli* non-ESBL, sensitive to ertapenem and meropenem. Cultures also grew *Staphylococcus haemolyticus *sensitive to vancomycin. Ertapenem was changed to meropenem 500 milligrams every 6 hours, and IV vancomycin 1 gram every 12 hours was added. The transrectal tube was delivering approximately 40 ml of purulent fluid per day, scant by day 14. 

**Figure 3:**
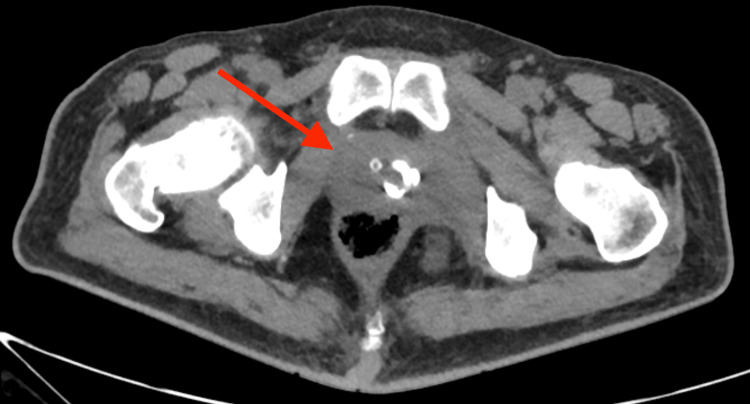
Follow-up non-contrast computed tomography; complete resolution of abscess; transrectal drainage catheter was removed soon afterward

On day 16 repeat CT scan demonstrated complete resolution of the PA (Figure 3), and IR removed his transrectal tube. A suprapubic cystostomy catheter was subsequently deployed (also by IR) with concomitant removal of his transurethral bladder catheter. We sought to divert urine to improve healing.

Infectious diseases service recommended four weeks of IV ertapenem and vancomycin antibiotics as an outpatient. The patient was discharged on day 20 of hospitalization. Insulin glargine 75 units at bedtime and insulin lispro 28 units pre-meal were continued regimens at discharge from our hospital, along with tamsulosin 0.4 mg daily.

The patient visited the urology clinic four weeks later; no complaints. Repeat urine cultures were negative. Voiding trial was successful, so the suprapubic catheter was removed. He received a total of 1 month of IV antibiotics.

## Discussion

PAs are rare. The disease peaks in the fifth and sixth decades in patients with urinary retention secondary to BPH and indwelling urinary catheters. PAs are usually caused by the reflux of infected urine contents into prostatic ducts. PA is a common complication from conditions such as acute and chronic bacterial prostatitis. They may arise from conditions associated with voiding dysfunction such as BPH and neurogenic bladder. Chronic medical conditions may contribute: uncontrolled diabetes mellitus, chronic renal failure, cirrhosis, conditions requiring chemotherapy or immunosuppressive therapy, and human immunodeficiency virus/acquired immunodeficiency syndrome [1]. 

Before antibiotics, PA was most commonly caused by gonorrheal infections and hematogenous spread of *Staphylococcus aureus* from osteomyelitis, furunculosis, and rheumatic fever. In the post-antibiotic era, gram-negative organisms predominate in PAs, especially *Escherichia coli*, which also predominates in acute prostatitis. Other gram-negative organisms such as *Klebsiella pneumoniae*, *Enterococcus* species, *Pseudomonas aeruginosa*, and* Brucella melitensis* have been reported. Reported gram-positive organism is *Staphylococcus aureus*. Fungal offenders include *Candida* species, *Cryptococcus neoformans*, *Blastomycosis dermatitidis,* and *Histoplasma capsulatum*. *Mycobacterium* species have also been reported [1,6].

Common presenting symptoms and classical signs of a PA are a mix of UTI and obstructive symptoms, such as dysuria, urgency, a sensation of incomplete voiding, perineal pain, and urinary retention [4]. Diagnosis is difficult, as patients can present with persistent fever despite the use of tailored IV broad-spectrum antibiotics, in which advanced imaging may be required to rule out prostatic abscesses in patients with risk factors for acute and chronic bacterial prostatitis (ABP and CBP). Pre- and post-contrast CT scans provide the diagnosis of prostatic abscess [2].

Transrectal ultrasound (TRUS) is the most common imaging technique for prostate abscess diagnosis, providing a diagnosis in 80%-100% of patients with prostate abscesses. It is contraindicated in patients with severe hemorrhoids and anal fistulas. CT scans can also provide the diagnosis but require pre- and post-IV contrast as areas with poor perfusion within the prostate can appear isodense on non-contrast images. CT scan has no clear advantage over transrectal ultrasound as the latter is more sensitive for visualizing intraprostatic abscesses. CT can be considered the initial imaging of choice in patients in which TRUS cannot be tolerated or contraindicated, such as patients with anal fistulas and anal hemorrhoids. MRI with and without IV contrast is superior to a CT scan but equally sensitive as TRUS in diagnosing PAs [1].

TRUS-guided aspiration has certain complications, such as infectious complications and rectourethral fistula formation. The incidence of infectious complications is low, but sepsis events have been described after the procedure, despite adequate abscess drainage. There is also the chance of incomplete abscess drainage or abscess recurrence. Abscess recurrence rates after aspiration have been reported to be up to 33% [1].

PAs may present with persistent fever of unknown origin nonresponsive to tailored broad-spectrum IV antibiotics, and/or acute urinary retention when the abscess is large enough to cause urinary tract flow obstruction. About half of the patients with PA will demonstrate positive urine culture. Results are often misleading as half of the urine culture results are different from abscess specimens [1]. For example in our case, urine cultures grew *Klebsiella pneumoniae* ESBL while PA cultures showed different organisms. Antibiotics should of course be tailored according to PA specimens. 

Standardized treatment guidelines are lacking. Admission to the hospital is warranted in immunocompromised patients. Treating PAs solely with antibiotics is accepted and effective for monofocal cavity PAs [1]. We would recommend empiric initial antibiotic therapy with IV vancomycin for methicillin-resistant *Staphylococcus aureus* (MRSA) coverage and IV broad spectrum gram-negative coverage with anti-pseudomonal coverage, such as IV piperacillin-tazobactam or IV cefepime, especially in immunocompromised patients. If there is clinical worsening or increased prostate abscess size in follow-up imaging, surgical drainage is warranted, in which we would tailor IV antibiotics according to prostate abscess drainage bacterial culture results. If the prostate abscess is <1 cm, sole antibiotic therapy is indicated for 4-6 weeks with serial imaging to ensure resolution and serial clinical follow-up to monitor for improvement or worsening. Surgical drainage is warranted in patients with abscesses larger than 1 cm, multiloculated PAs, or PAs which fail to respond to IV antibiotics. Incision and drainage (I&D) are often chosen. I&D can be performed promptly in a surgical suite or bedside under local anesthesia with low morbidity and can be repeated if necessary [4,7]. 

Different ultrasound-guided approaches have been described in the literature. Transrectal ultrasound-guided aspiration and/or tube drainage can also be performed promptly at the bedside and repeated in cases of recurrence or persistence. The incidence of infectious complications after drainage is low and 89% success rates are reported [1,8]. Another single-center retrospective study of 150 consecutive patients with symptomatic pelvic fluid collections (SPFCs) showed a 95% technical success rate with drainage using ultrasound and coadjuvant fluoroscopy [9]. In our case, no fluoroscopy guidance was utilized.

*Staphylococcal aureus* infections are increasingly encountered in prostate abscesses in the post-antibiotic era, particularly in immunocompromised patients [10]. *Staphylococcus haemolyticus* has never been reported as a gram-positive bacterial etiology of a prostatic abscess, despite its known notoriety in CBP [1,3]. We strongly believe this observation is important. PAs are typically treated empirically only with gram-negative antibiotic coverage such as broad-spectrum fluoroquinolones [11]. This case suggests that providers should consider adding empiric broad-spectrum antibiotics that also cover gram-positive organisms such as *Staphylococcus haemolyticus* and *Staphylococcus aureus*.

Our case presents an elderly patient with risk factors for a PA (poorly controlled diabetes mellitus and BPH) presenting with sepsis. Surprisingly, PA abscess cultures grew *Staphylococcus haemolyticus*, a gram-positive, coagulase-negative coccus that colonizes skin flora. *Staphylococcus haemolyticus* occupied PA in our patient. This is perhaps explained by an undiagnosed acute prostatitis that progressed to chronic bacterial prostatitis. ABP and CBP are risk factors for the development of PA [4]. In 2017, Ebrahimpour et al. reported a case of CBP secondary to *Staphylococcus haemolyticus* [12]. 

Our patient shared common risk factors for acquiring an ABP, such as anatomical anomalies predisposing to urogenital infections and a history of chronic indwelling and intermittent bladder catheterizations. Our patient was also at risk for the progression of ABP to CBP: previous manipulations with urinary catheterizations and diabetes. Prostatitis symptoms often overlap with BPH [13]. 

*Staphylococcus haemolyticus* is known to be part of the microbiome. Its role as a pathogen may be underestimated. Clinical context can help to differentiate between contaminant versus pathogen. When it causes disease, it is frequently associated with an immunocompromised state and implanted devices [14]. In our patient, uncontrolled diabetes mellitus caused immune compromise. Repeated catheterizations further potentiated infection.

## Conclusions

Prostatic abscesses (PAs) are rare. Mortality rates are as high as 16%. Diagnosis requires clinical suspicion, especially in patients with persistent sepsis of unknown source despite treatment with broad-spectrum antibiotics in a male with associated risk factors. *Staphylococcus haemolyticus* can be a causative organism of a PA. Clinicians should be aware of the increase in *Staphylococcus* species causing PAs. Broad-spectrum empiric antibiotic coverage for PA should include gram-positive and gram-negative broad-spectrum coverage before tailoring antibiotics accordingly with procedural drainage cultures. Constant monitoring and long-term follow-up are warranted during treatment, as PA patients can rapidly progress to worsening sepsis and death, especially in immunocompromised patients. Also, there can be persistent PA collections in unsuccessful surgical drainage procedures, resulting in a relapse of infection and sepsis, which may necessitate repeating surgical drainage procedures. In general, antibiotic treatment is an adjunct to treatment, as most patients with PA will require drainage. Transrectal ultrasound-guided drainage is a prompt and effective minimally invasive surgical treatment option for PA and can be performed without fluoroscopy.
